# First-degree relatives of cancer patients: a target group for primary prevention? A cross-sectional study

**DOI:** 10.1038/s41416-018-0057-2

**Published:** 2018-03-21

**Authors:** Ulrike Haug, Oliver Riedel, Constanze Cholmakow-Bodechtel, Louise Olsson

**Affiliations:** 1 Department of Clinical Epidemiology, Leibniz Institute for Prevention Research and Epidemiology – BIPS, Achterstr. 30, 28359 Bremen, Germany; 2 Faculty of Human and Health Sciences, University of Bremen, Grazer Straße 2, 28359 Bremen, Germany; 3 Department of Public Health and Epidemiology, Kantar Health GmbH, Landsberger Str. 284, 80687 Munich, Germany; 4 Department of Molecular Medicine and Surgery, Karolinska Institutet, Karolinska University Hospital, Solna (L1:00), 171 76 Stockholm, Sweden

**Keywords:** Risk factors, Cancer prevention, Lifestyle modification, Cancer prevention, Epidemiology

## Abstract

**Background:**

Persons with a first-degree relative (FDR) with cancer are at increased cancer risk. We investigated preventive behaviour, cancer risk perception and readiness to change an unhealthy lifestyle in persons with and without an FDR with cancer.

**Methods:**

Using an online questionnaire, we conducted a cross-sectional study in Germany including persons (≥35 years) with an FDR with colorectal, lung, prostate, breast, stomach or cervical/uterine cancer (*n* = 621) and persons without cancer in FDRs (*n* = 303). Quota sampling ensured similar age and sex distributions in both groups.

**Results:**

Unfavourable lifestyle factors were equally common in both groups. The proportion perceiving an increased cancer risk significantly differed (*p* < 0.0001) with 4% among respondents without cancer in FDRs and 18% (colorectal cancer) to 30% (stomach cancer) among cancer patients’ relatives. The proportion of smokers ready to quit smoking was significantly higher among those perceiving an increased vs. a lower cancer risk (64 vs. 46%, *p* = 0.04). There was a similar association for readiness to increase physical activity and consumption of fruits/vegetables and to reduce alcohol consumption.

**Conclusions:**

Given the increased risk perception and motivation to change an unhealthy lifestyle, our study provides a strong rationale for research on the effectiveness of lifestyle interventions in cancer patients’ relatives.

## Introduction

Cancer is one of the leading causes of premature mortality in many parts of the world.^1^ It is generally accepted that a relevant proportion of cancers is attributable to an unhealthy lifestyle and could thus be prevented.^2,3^ In practice, however, effecting change in preventive behaviour is a major challenge. Persons with a family history of cancer (FHC) form an important target group for cancer prevention given their increased cancer risk attributable to both genetic and environmental factors as well as interactions between both.^4,5^

Cancer risk perception has been suggested as one of the key factors associated with health behaviour change.^6^ It is thus of particular interest whether persons with a familial cancer risk actually perceive this risk and are more motivated to change an unhealthy lifestyle. However, not much is known in this field. A systematic review published in 2011 on cancer risk perception showed that the majority of pertinent studies—mainly conducted in the U.S.—focused on subjects at increased risk of breast cancer and primarily investigated the psychology of and factors associated with cancer risk perception.^7^ Although understanding determinants of cancer risk perception is important, key questions relevant to the rationale of preventive strategies based on FHC are still unanswered.

To contribute to this research field, we aimed to compare cancer risk perception in various groups with an FHC and in a control group without an FHC and to explore its relevance regarding the motivation to change an unhealthy lifestyle. We hypothesised that respondents who perceived a higher than average risk for developing cancer were more often ready to change an unhealthy lifestyle as compared to respondents who perceived their cancer risk as lower than average.

## Materials and Methods

### Study design

We conducted a cross-sectional study in Germany in 2012 using an anonymous online questionnaire. The questionnaire was administered via e-mail by Kantar Health GmbH (Munich, Germany), a healthcare-focused consultancy with experience in implementing health surveys in Germany,^8–10^ to a population-based sample of persons who had agreed to regularly participate in online surveys. After being informed about the study, the persons could accept or decline participation. The information was kept general to avoid selection effects, e.g., regarding lifestyle factors or cancer risk perception. The study was exempted from institutional review board review by the Ethics Committee of the Medical Faculty of Heidelberg University as no identifying data were collected.

We only included individuals ≥35 years because beginning at this age (1) an FHC is increasingly common and (2) preventive measures such as biennial medical check-ups are offered in Germany. The project allowed for including 1000 individuals. We used a quota sampling approach to ensure that the study population comprised reasonably sized groups with and without an FHC. Specifically, we set quota to include 300 individuals in whom no first-degree relative (FDR) had been diagnosed with cancer and 700 individuals in whom an FDR had been diagnosed with one of the following cancers: colorectal, lung, prostate, breast, stomach or cervical/uterine cancer. These cancers were selected to include the three cancers most common in Germany in men and women, as well as less common cancers for comparison (stomach and cervical/uterine cancer).^11^ Given that the German lay terms for cervical and uterine cancer are rather identical, we asked for both cancers combined as it seems unlikely that relatives of such patients could reliably distinguish one from the other. We also set quotas on age and sex to ensure population representativeness as well as comparability between the subgroups with and without an FDR with cancer in this regard. Once the quota criteria were met, respondents with the respective characteristics were branched to a quota complete screen. Respondents younger than 35 years or who reported cancer diagnoses among FDRs not relevant to the survey were screened out.

### Questionnaire

The questionnaire asked for the number and sex of FDRs (biological parents, siblings and children) and whether any of them had ever been diagnosed with cancer. Furthermore, information on sociodemographic characteristics, anthropometric measures, lifestyle factors (smoking, physical activity, consumption of alcohol, fruits and vegetables, meat) and pre-existing diseases (e.g., cancer) was collected. The tools used to collect information on lifestyle factors are described in Supplement 1. For each lifestyle factor, the readiness to change was assessed with a seven-point Likert scale (see Supplement 2). Respondents without a pre-existing cancer diagnosis were asked how they estimated their personal risk of developing cancer compared to the average risk among people of the same age and sex using a five-point Likert scale (see Supplement 2). The online questionnaire was pre-tested by persons across the age range of the target population.

### Data analyses

Given that our research question was on cancer risk perception, we excluded respondents reporting a pre-existing cancer diagnosis. We calculated individual weighting factors to account for deviations between included respondents and the general population with respect to age, sex, education and region. In all analyses, we used the individual weighting factors to optimise representativeness of the results. We first described the study population overall and stratified by FHC. In a next step, we focused on respondents in one or more of the least favourable categories regarding lifestyle factors and BMI (body mass index). That is, we considered current smokers, respondents in the highest category regarding alcohol consumption or the consumption of red and/or processed meat, respondents in the lowest category regarding the consumption of fruits and/or vegetables or regarding physical activity, as well as respondents with a BMI ≥30 kg/m^2^. We described readiness to change the respective factor for each of these subgroups (e.g., the intention to quit smoking for current smokers). We then assessed the association between readiness to change the respective factor ('ready' vs. 'not ready') and perceived cancer risk ('lower or much lower than average' vs. 'higher or much higher than average') (see Supplement 1). This was to test our hypothesis that respondents who perceived a higher or much higher than average risk for developing cancer were more often ready to change the respective factor as compared to respondents who perceived their cancer risk as lower or much lower than average. For comparisons of groups, we used chi-square tests for categorical variables. All statistical analyses were carried out using SAS software, version 9.3 (SAS Institute Inc., Cary, NC).

## Results

Overall, we included 924 respondents in the analysis (see Supplement 3). There were 621 respondents with an FDR diagnosed with cancer and 303 respondents without. Among the former, respondents with an FDR with colorectal cancer were the largest group (*N* = 173), followed by respondents with an FDR with breast cancer (*N* = 163), with lung cancer (*N* = 152), with cervical or uterine cancer (*N* = 106), with prostate cancer (*N* = 94) and with stomach cancer (*N* = 75). There were 142 respondents with more than one of these cancer diagnoses in FDRs. The median time since diagnosis of the FDR was 19 years (interquartile range: 10–31 years) regarding parents with cancer and 10 years (interquartile range: 4–19 years) regarding siblings or children with cancer.

Table 1 provides information on sociodemographic characteristics, lifestyle behaviours and BMI among respondents.

**Table 1:** Sociodemographic characteristics, lifestyle behaviours and BMI overall and according to family history of cancer

	All	No FDR with cancer	FDR with cancer	FDR with colorectal cancer	FDR with lung cancer	FDR with prostate cancer	FDR with breast cancer	FDR with cervical or uterine cancer	FDR with stomach cancer
	*N* = 924	*N* = 303	*N* = 621^a^	*N* = 173	*N* = 152	*N* = 94	*N* = 163	*N* = 106	*N* = 75
*Sex*									
Female, *N* (%)	478 (52)	159 (52)	319 (51)	100 (58)	80 (53)	41 (44)	84 (52)	61 (58)	38 (51)
Male, *N* (%)	446 (48)	144 (48)	302 (49)	73 (42)	72 (47)	53 (56)	79 (48)	45 (42)	37 (49)
*Age*									
Mean (years)	55.2	55.1	55.3	57.3	55.3	55.0	54.2	53.7	56.7
35–44 years, *N* (%)	195 (21)	77 (25)	118 (19)	21 (12)	30 (20)	15 (16)	36 (22)	27 (25)	13 (17)
45–54 years, *N* (%)	259 (28)	69 (23)	190 (31)	48 (28)	43 (28)	36 (38)	54 (33)	33 (31)	18 (24)
55–64 years, *N* (%)	199 (22)	59 (19)	140 (23)	49 (28)	34 (22)	20 (21)	32 (20)	17 (16)	20 (27)
65–74 years, *N* (%)	237 (26)	87 (29)	150 (24)	49 (28)	40 (26)	19 (20)	37 (23)	25 (24)	21 (28)
≥75 years, *N* (%)	34 (4)	11 (4)	23 (4)	6 (3)	5 (3)	4 (4)	4 (2)	4 (4)	3 (4)
*Years of schooling*									
≤9 years, *N* (%)	199 (22)	70 (23)	129 (21)	44 (25)	41 (27)	19 (20)	29 (18)	22 (21)	19 (25)
10–11 years, *N* (%)	395 (43)	123 (41)	272 (44)	74 (43)	72 (47)	35 (37)	66 (40)	49 (46)	29 (39)
≥12 years, *N* (%)	330 (36)	110 (36)	220 (35)	55 (32)	39 (26)	40 (43)	68 (42)	35 (33)	27 (36)
*Vocational training*									
None, *N* (%)	41 (4)	15 (5)	26 (4)	7 (4)	8 (5)	4 (4)	5 (3)	8 (8)	6 (8)
Non-academic, *N* (%)	618 (67)	207 (68)	411 (66)	118 (68)	106 (70)	56 (60)	110 (67)	69 (65)	46 (61)
Academic, *N* (%)	264 (29)	81 (27)	183 (30)	48 (28)	37 (25)	34 (36)	48 (29)	29 (27)	23 (31)
*Smoking status*									
Current, *N* (%)	274 (30)	78 (26)	196 (32)	54 (31)	57 (38)	27 (29)	51 (31)	32 (30)	23 (31)
Former, *N* (%)	246 (27)	83 (27)	163 (26)	49 (28)	35 (23)	25 (27)	47 (29)	24 (23)	19 (25)
Never, *N* (%)	404 (44)	142 (47)	262 (42)	70 (40)	60 (39)	42 (45)	65 (40)	50 (47)	33 (44)
*Alcohol consumption, past 12 months*									
Once a month or less, *N* (%)	394 (43)	128 (42)	266 (43)	67 (39)	62 (41)	44 (47)	69 (42)	53 (50)	26 (35)
2–4 times a month, *N* (%)	197 (21)	67 (22)	130 (21)	42 (24)	35 (23)	17 (18)	26 (16)	19 (18)	20 (27)
Twice a week or more, *N* (%)	333 (36)	108 (36)	225 (36)	64 (37)	55 (36)	33 (35)	68 (42)	34 (32)	29 (39)
*Consumption of fruits and/or vegetables, past 12 months*									
Less than once a day, *N* (%)	208 (23)	84 (28)	124 (20)	34 (20)	31 (20)	17 (18)	34 (21)	25 (24)	13 (17)
Daily, but <4 times a day, *N* (%)	511 (55)	162 (53)	349 (56)	101 (58)	83 (55)	55 (59)	91 (56)	60 (57)	42 (56)
Four times a day or more, *N* (%)	205 (22)	57 (19)	148 (24)	38 (22)	38 (25)	22 (23)	38 (23)	21 (20)	20 (27)
*Consumption of red and/or processed meat, past 12 months*									
Once a week or less, *N* (%)	99 (11)	30 (10)	69 (11)	18 (10)	20 (13)	15 (16)	21 (13)	10 (9)	5 (7)
2–7 times a week, *N* (%)	681 (74)	225 (74)	456 (73)	135 (78)	108 (71)	65 (69)	120 (74)	76 (72)	52 (69)
Twice a day or more, *N* (%)	144 (16)	48 (16)	96 (15)	20 (12)	24 (16)	14 (15)	22 (14)	20 (19)	18 (24)
*Physical activity for at least 30 min, past 12 months*									
Less than twice a week, *N* (%)	320 (35)	114 (38)	206 (33)	65 (38)	42 (28)	38 (40)	53 (33)	26 (25)	29 (39)
2–4 times a week, *N* (%)	405 (44)	124 (41)	281 (45)	80 (46)	77 (51)	38 (40)	67 (41)	51 (48)	31 (41)
5 times a week or more, *N* (%)	199 (22)	65 (21)	134 (22)	28 (16)	33 (22)	18 (19)	43 (26)	29 (27)	15 (15)
*Body mass index*									
<22.5 kg/m^2^, *N* (%)	148 (16)	52 (17)	96 (15)	30 (17)	25 (16)	16 (17)	22 (14)	17 (16)	13 (17)
22.5–24.9 kg/m^2^, *N* (%)	175 (19)	69 (23)	106 (17)	32 (19)	27 (18)	15 (16)	27 (17)	20 (19)	12 (16)
25–29.9 kg/m^2^, *N* (%)	334 (36)	103 (34)	231 (37)	55 (32)	62 (41)	34 (36)	69 (42)	37 (35)	25 (33)
≥30.0 kg/m^2^, *N* (%)	267 (29)	79 (26)	188 (30)	56 (32)	38 (25)	29 (31)	45 (28)	32 (30)	25 (33)

*BMI* body mass index, *FDR* first-degree relative

^a^Less than the sum of cancer-specific subgroups as there were respondents reporting more than one relevant cancer diagnosis in FDRs

Unfavourable lifestyle factors and a high BMI were equally common in respondents with and without an FDR with cancer. The proportion of smokers was higher in respondents with an FDR with lung cancer as compared to respondents without an FDR with cancer (38 vs. 26%, *p* = 0.012). Low consumption of fruits and vegetables tended to be more common in respondents without vs. with an FDR with cancer, but this difference was not statistically significant (Table 1).

**Fig. 1:**
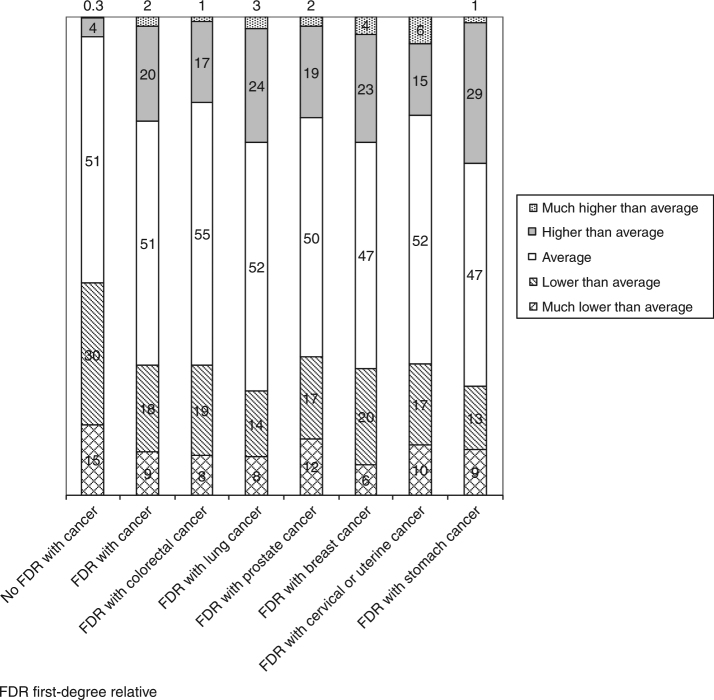
Distribution of the respondents’ answers (stratified by family history of cancer) to the question how they estimate their personal risk of developing cancer compared to the average risk among people of the same age and sex (numbers indicate percentages)

Figure 1 shows the distribution of answers regarding cancer risk perception. About half of the respondents across all subgroups perceived their risk as 'average', i.e., similar to the average risk among people of the same age and sex. The proportion of respondents perceiving their cancer risk as higher or much higher than average was 4% in the subgroup without an FDR with cancer and ranged between 18% (FDR with colorectal cancer) and 30% (FDR with stomach cancer) in the remaining subgroups.

**Fig. 2:**
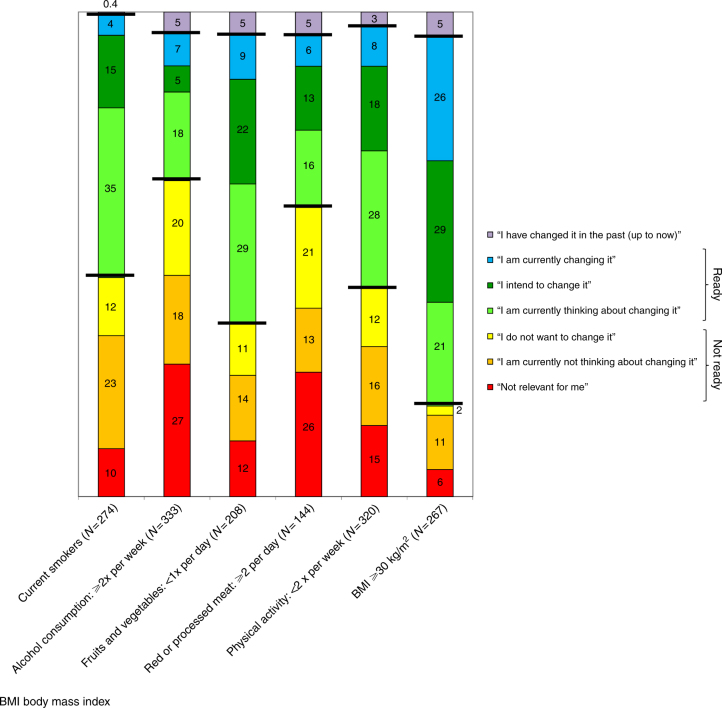
Distribution of answers regarding readiness to change among respondents with an unhealthy lifestyle factor or an elevated BMI (numbers indicate percentages)

Figure 2 illustrates readiness to change among respondents with an unhealthy lifestyle factor or an elevated BMI. Among smokers, 54% reported currently thinking about quitting, intending to quit or being about to quit smoking (group 'ready'). Among respondents with a low consumption of fruits and vegetables, 60% were in the group 'ready' with respect to increasing consumption of fruits and vegetables. Among respondents with a BMI equal or above 30 kg/m^2^, 76% were in the group 'ready' with respect to lowering the BMI. The proportion of those 'ready' to change the respective factor was lowest among respondents with a high consumption of alcohol and among respondents with a high consumption of red or processed meat (30 and 35%, respectively).

Table 2 shows readiness to change the respective factor according to perceived cancer risk. Among current smokers, the proportion of respondents ready or about to quit smoking was 18% higher in those perceiving a higher than average cancer risk as compared to those perceiving a lower than average cancer risk (*p* = 0.04). Similarly, readiness to change the respective factor was more common in those perceiving an increased risk of cancer among respondents with a high alcohol consumption, among respondents with a low consumption of fruits and vegetables and among respondents with a low level of physical activity (*p* < 0.05).

**Table 2:** Readiness to change an unhealthy lifestyle according to perceived cancer risk

Perceived cancer risk	'Ready' to change^a^			*p* value^b^
*Current smokers*				
	Overall, *N* = 273	Of these, 'ready' to quit smoking, *N* (row-%)		
Lower or much lower than average, *N*	52	24 (46%)		*p* = 0.04
Average, *N*	140	73 (52%)		
Higher or much higher than average, *N*	81	52 (64%)		
*Alcohol consumption: ≥2 × per week*				
	Overall, *N* = 318	Of these, 'ready' to reduce alcohol consumption, *N* (row-%)		
Lower or much lower than average, *N*	103	27 (26%)		*p* = 0.02
Average, *N*	163	51 (31%)		
Higher or much higher than average, *N*	52	23 (44%)		
*Fruits and vegetables: <1 × per day*				
	Overall, *N* = 198	Of these, 'ready' to increase consumption of fruits and vegetables, *N* (row-%)		
Lower or much lower than average, *N*	52	29 (56%)		*p* = 0.04
Average, *N*	107	65 (61%)		
Higher or much higher than average, *N*	39	30 (77%)		
*Red or processed meat: ≥2 × per day*				
	Overall, *N* = 137	Of these, 'ready' to reduce consumption of red or processed meat, *N* (row-%)		
Lower or much lower than average, *N*	33	12 (36%)		*p* = 0.73
Average, *N*	76	30 (39%)		
Higher or much higher than average, *N*	28	9 (32%)		
*Physical activity: <2 × per week*				
	Overall, *N* = 310	Of these, 'ready' to increase physical activity, *N* (row-%)		
Lower or much lower than average, *N*	101	50 (50%)		*p* = 0.08
Average, *N*	161	92 (57%)		
Higher or much higher than average, *N*	48	31 (65%)		
*BMI ≥ 30 kg*/*m*^2^				
	Overall, *N* = 267	Of these, 'ready' to lower BMI, *N* (row-%)		
Lower or much lower than average, *N*	61	48 (79%)		*p* = 0.42
Average, *N*	146	116 (79%)		
Higher or much higher than average, *N*	46	39 (85%)		

*BMI* body mass index

^a^Respondents who think about changing, intend to change or are currently changing the respective factor

^b^The *p* value refers to the comparison of the proportion that is 'ready' to change between those perceiving a lower or much lower than average risk of cancer vs. those perceiving a higher or much higher than average risk

## Discussion

Our study provides several insights into risk factor profiles, risk perception and motivation to change an unhealthy lifestyle in well-defined subgroups of persons with an FDR with cancer and persons without such a family history. Our findings showed that perceiving an increased cancer risk (i) was considerably more common among persons with vs. without an FDR with cancer (18–30 vs. 4%), which held true for all cancer diagnoses studied and (ii) coincides with a significantly higher motivation to change an unhealthy lifestyle. Combining both findings suggests that targeted preventive measures for persons with an FDR with cancer might be particularly promising in view of both the increased risk and the increased risk perception. In other words, preventive measures may be more likely to fall on fruitful soil in this target group as compared to the general population, where low compliance and self-selection according to lower risk often are critical issues limiting effectiveness.

To the best of our knowledge, there is no similarly designed study to which we could directly compare our findings. Previous studies among persons with an FHC mostly focused on only one of the aspects addressed in our study (e.g., either risk perception or lifestyle factors or readiness to change), but did not investigate them coherently in one study population. Furthermore, they often focused on a specific FHC (e.g., breast cancer) or only considered any but not specific FDRs, while we considered FDRs of various cancer patients in parallel.

An association between any FHC and a higher perceived cancer risk has been shown previously.^12,13^ For example, a study based on data from the National Health Interview Survey compared various established risk factors regarding their impact on cancer risk perception and identified FHC as the most influential determinant.^13^ Associations regarding a specific family history and a higher perceived cancer risk have mainly been investigated and shown for breast cancer and to a lesser degree also for other cancers.^7^ Acheson et al. compared the magnitude of the effect of family history on perceived risk of coronary heart disease, stroke, diabetes, and breast, ovarian, and colon cancers and found the highest effect for colon and breast cancer.^14^

Regarding the association between lifestyle habits and FHC, our study did not show any differences in lifestyle characteristics associated with FHC, with some exceptions, e.g., a higher proportion of smokers in respondents with an FDR with lung cancer. This is consistent with studies from the United States that showed lifestyle behaviour to be largely unrelated to family history of breast cancer,^15,16^ ovarian cancer^16^ and colorectal cancer.^15,16^ In these studies, some characteristics also tended to be less favourable in persons with an FHC, such as a higher proportion of overweight persons among FDRs of colorectal cancer patients.^16^ Another study from the United States found a higher proportion of smokers among women with a family history of breast cancer.^17^ A study from Japan found a slightly higher proportion of smokers among women with vs. without a family history of uterine cancer.^18^

Available evidence thus suggests that persons with an FHC do not have a healthier, but partly an unhealthier lifestyle as compared to persons without an FHC. On the other hand, a study by Lemon et al.^19^ found that 42% of women with an FDR with breast cancer reported any health behaviour change in the 6 months following the diagnosis of their relative. While this study supports the presence of a teachable moment in FDRs of breast cancer patients, it seems that this self-initiated health behaviour change shortly after diagnosis is only temporary and not a general phenomenon. Otherwise, our and other survey data would have shown more favourable lifestyle characteristics among persons with a family history of breast cancer. Thus, research into educational interventions that support a permanent change in health behaviour presents an important aspect to take full advantage of this teachable moment.

The motivation to change an unhealthy lifestyle among persons with an FHC has also been supported by a study conducted within the Colon Cancer Family Registry. It showed that 81% of 401 persons with two or more relatives diagnosed with colorectal cancer were willing to take part in a lifestyle programme and there was a significant correlation between a higher level of concern about cancer and an inclination to participate in these programmes.^20^ In another study, 81% of smokers with a family history of lung cancer considered or planned to quit smoking (contemplation or preparation phase), while this proportion was about 13% lower in smokers without a family history of lung cancer.^21^

Despite the rationale, there is a lack of interventional studies investigating the effectiveness of health behaviour interventions among relatives of cancer patients.^22^ The Family Healthware Impact trial provided sex- and age-specific health messages to persons with a family history of stroke, heart disease, diabetes, breast cancer, ovarian cancer and colorectal cancer. After 6 months, participants in the intervention group were significantly more likely to have increased their daily fruits and vegetable intake and their physical activity than the control group.^23^ Given that risk perception is an important mediator, which was most pronounced in relatives of cancer patients, the effectiveness may have been highest in these persons but the study was not designed to conduct such subgroup analyses.^24,25^

Further interventional studies among FDRs of cancer patients are thus needed. In the design of such intervention studies, the strategy on how to reach the target population is an important aspect, e.g., regarding the optimal timing of the intervention. Receptivity to prevention efforts may be highest if family members were approached closely to the time of diagnosis of their relative. This would also mean that our study even underestimated the motivation to change an unhealthy lifestyle given that all respondents were surveyed at one point in time, i.e., the cancer diagnosis of their relative may have been long past. Furthermore, risk communication would be an important component in the design of such intervention studies given the central role of risk perception as a mediator regarding behaviour change. The fact that the majority of persons in all subgroups perceived an average or lower than average cancer risk is a general phenomenon known as unrealistic optimism about susceptibility to health problems.^26^ It remains to be clarified whether risk communication strategies can reduce this unrealistic optimism and thus further increase the potential of preventive measures in FDRs of cancer patients.

A substantial proportion of individuals in a population experience a cancer diagnosis among relatives during their lifetime. Even if only some of these individuals could be motivated to change an unhealthy lifestyle, the impact on population health may be considerable given the many beneficial effects of primary prevention on various health outcomes. This may complement and exceed the potential of cancer screening interventions, where the benefit is limited to specific cancer sites.

The following limitations of our study should be considered. First, given the research focus and cross-sectional design of our study, we did not use our data to investigate determinants of cancer risk perception. That research question would require a different study design. For example, although available we did not consider information on screening history such as prior use of colonoscopy or mammography in our analyses. Such examinations could influence cancer risk perception in opposite directions depending on the respective findings. Interpretation is thus complex and not relevant to our objective, which was to investigate the current status of cancer risk perception in the study population from a cross-sectional perspective and not its determinants. Second, different methods to assess cancer risk perception have been suggested, but there is no gold standard. We therefore decided to use a simple and pragmatic tool previously tested in other studies.^14^ Although not feasible in this study, in-depth interviews on perceptions of cancer risk and control provide important information on these psychological constructs and their correlation.^27^ Third, although we included 700 respondents with an FHC overall and restricted the types of cancer, some subgroups had a rather limited sample size. Fourth, information on FHC was collected by self-report. While validity of this self-reported information is not perfect and varies between cancers,^28^ this limitation is less relevant for studies on cancer risk perception as compared to studies on the aetiology of cancer. Finally, volunteer bias can never be ruled out completely but we tried to ensure representativeness of our findings by using population-representative quota on age and sex and additionally by calculating individual weighting factors to account for deviations between included respondents and the general population with respect to age, sex, education and region.

In conclusion, given the increased risk perception and the concurrent higher motivation among relatives of cancer patients to change an unhealthy lifestyle, our study provides a strong rationale for conducting interventional research to assess the effectiveness of lifestyle interventions in this risk group. In the long run, the impact on population health may be considerable given the size of the target population and the many beneficial effects of primary prevention on various health outcomes.

### Availability of data and material

The data sets used and/or analysed during the current study are available from the corresponding author on reasonable request.

## Electronic supplementary material

Supplemental material(DOCX 51 kb)

Supplemental material
