# Supplementary material for: First-degree relatives of cancer patients: a target group for primary prevention? A cross-sectional study
Source: Br J Cancer. 2018 Mar 21;118(9):1255–61. doi: 10.1038/s41416-018-0057-2 (PMC5943415; doi:10.1038/s41416-018-0057-2)
Supplement: Supplementary file 1 — Supplemental material(DOCX 51 kb) [file 41416_2018_57_MOESM1_ESM.docx]

**Supplementary material:**

**Supplement 1:** Description of the tools used to collect information on lifestyle factors

**Supplement 2:** Collection and categorisation of information on readiness to change and risk perception.

**Supplement 3:** Flow chart illustrating the inclusion and exclusion of respondents.
