# Supplementary material for: First-degree relatives of cancer patients: a target group for primary prevention? A cross-sectional study
Source: Br J Cancer. 2018 Mar 21;118(9):1255–61. doi: 10.1038/s41416-018-0057-2 (PMC5943415; doi:10.1038/s41416-018-0057-2)
Supplement: Supplementary file 2 — Supplemental material [file 41416_2018_57_MOESM2_ESM.docx]

**Supplementary Material**

**Supplement 1:** Description of the tools used to collect information on lifestyle factors

*General note:* The primary aim of the questions on lifestyle risk factors in our study was to distinguish persons who are the least adherent to current recommendations for cancer prevention from persons who adhere to a larger degree to these recommendations (see Table 1). Information beyond this purpose was not collected in order to keep the questionnaire short. We therefore used short forms of standard tools for collecting information on lifestyle factors, skipping questions that are only important to exactly quantify lifestyle factors (e.g. questions required to calculate grams of alcohol, pack-years of smoking, grams of daily consumption of certain food ingredients, etc.) as this information was not needed for our study.

1. Smoking

The first question (“Have you ever smoked more than 100 cigarettes in your life?”) aimed to distinguish between never smokers and past/current smokers. We used the common limit of 100 cigarettes in a lifetime to identify those individuals who had never smoked at all or never progressed beyond being an experimental smoker. Those who answered the first question with “yes” were asked if they had ever smoked regularly (daily for at least one year), when they had started smoking, how many years they had smoked regularly and if they smoked currently. If the latter was answered with “yes”, the number of cigarettes per day was asked for and if the answer was “no” the stopping age was asked for. Studies assessing the validity of self-reported smoking behaviour (e.g. by comparison of self-reported smoking with cotinine levels) concluded that it provides accurate estimates of the smoking status (Studts *et al*, 2006; Wong *et al*, 2012).

1. Alcohol consumption

Persons were asked how often they usually drink alcoholic beverages such as wine, beer, schnapps, liqueur or cocktails. Six answering options were offered (never, once per month or less, 1-4 times per month, 2-3 times per week, 4-6 times per week, daily). Given that the survey was completed within four weeks (May / June) heterogeneity due to seasonal variation was not relevant. Studies assessing validity of self-reported drinking behaviour found a similar distribution between self- versus nonself-reports as regards moderate drinking and usual frequency of drinking, which represents the information that was important in our study (Bongers *et al*, 1999). There seems to be a discordance in self-reported versus nonself-reported "heavy" drinking (Bongers *et al*, 1999), but this category was not important in our study as for cancer prevention any alcohol consumption should be avoided.

1. Consumption of fruits and vegetables, and of red or processed meat

We used a short form of the EPIC food frequency questionnaire (Bohlscheid-Thomas *et al*, 1997). Persons were amongst others asked about their average consumption of

- Sausage / processed meat
- Poultry
- Other meat (beef, pork, sheep)
- Fish
- Fruits / vegetables
- Vegetables / salad

For each of these categories, eight answering options were offered (never, less than once per month, 1-3 times per month, 1-3 times per week, 4-6 times per week, once per day, 2-3 times per day, 4-5 times per day). Given that the survey was completed within four weeks (May / June) heterogeneity due to seasonal variation was not relevant.

1. Physical activity

The questions on physical activity were inspired by the validated International Physical Activity Questionnaire Short Form (IPAQ-SF) (Craig *et al*, 2003), but were further shortened as the calculation of metabolic equivalents was not required for this study.

Persons were asked how many days per week they engage in physical activities that accelerate the pulse, make breathing harder or lead to sweating. They were then further asked how much time they usually spend on these physical activities (hours/minutes per day). Given that the survey was completed within four weeks (May / June) heterogeneity due to seasonal variation was not relevant.

References Supplement 1:

Bohlscheid-Thomas S, Hoting I, Boeing H, Wahrendorf J (1997) Reproducibility and relative validity of food group intake in a food frequency questionnaire developed for the German part of the EPIC project. European Prospective Investigation into Cancer and Nutrition. *Int J Epidemiol* **26 Suppl 1**: S59-70

Bongers IM, van de Goor IA, Garretsen HF, van Oers HA (1999) Aggregate comparisons of self-reported versus nonself-reported drinking in a general population survey. *Subst Use Misuse* **34**(3): 421-41

Craig CL, Marshall AL, Sjostrom M, Bauman AE, Booth ML, Ainsworth BE, Pratt M, Ekelund U, Yngve A, Sallis JF, Oja P (2003) International physical activity questionnaire: 12-country reliability and validity. *Med Sci Sports Exerc* **35**(8): 1381-95

Studts JL, Ghate SR, Gill JL, Studts CR, Barnes CN, LaJoie AS, Andrykowski MA, LaRocca RV (2006) Validity of self-reported smoking status among participants in a lung cancer screening trial. *Cancer Epidemiol Biomarkers Prev* **15**(10): 1825-8

Wong SL, Shields M, Leatherdale S, Malaison E, Hammond D (2012) Assessment of validity of self-reported smoking status. *Health Rep* **23**(1): 47-53

**Supplement 2:** Collection and categorisation of information on readiness to change and risk perception.

1. Readiness to change

For each lifestyle factor (smoking, physical activity, consumption of alcohol, fruits and vegetables, meat), the readiness to change was assessed with a seven-point Likert scale, offering the following options to answer:

*“not relevant for me”,*

*“I do not want to change it”,*

*“I am currently not thinking about changing it”,*

*“I am currently thinking about changing it”,*

*“I intend to change it”,*

*“I am currently changing it”,*

*“I have changed it in the past (up to now)”*.

When we assessed the association between readiness to change the respective factor and perceived cancer risk, we grouped the respondents into two groups regarding readiness to change the respective factor:

Group “Not ready”:

*“Not relevant for me”,*

*“I do not want to change it”,*

*“I am currently not thinking about changing it”*.

Group “Ready”:

*“I am currently thinking about changing it”,*

*“I intend to change it”,*

*“I am currently changing it*”.

We excluded respondents reporting that they had changed it in the past.

1. Risk perception

Respondents who did not report previously having been diagnosed with cancer were asked how they estimated their personal risk of developing cancer compared to the average risk among people of the same age and sex. A five-point Likert scale offered the following options:

“*much lower than average*”,

*“lower than average”,*

*“average”,*

*“higher than average”,*

*“much higher than average”*.

When we assessed the association between readiness to change the respective factor and perceived cancer risk, we considered the following groups:

Group 1:

*“much lower than average”,*

*“lower than average”.*

Group 2:

*“higher than average”,*

*“much higher than average”.*

We excluded respondents perceiving an “average” cancer risk.

We hypothesized that those respondents who perceived a higher or much higher than average risk for developing cancer (group 2) were more often ready to change the respective factor as compared to respondents who perceived their cancer risk as lower or much lower than average (group 1).

**Supplement 3:** Flow chart illustrating the inclusion and exclusion of respondents.

Without a first degree relative with cancer:

N=303

With a first degree relative with cancer:

N=621

Pre-existing cancer diagnosis:

N=88

Quota full:

Respondents were excluded because the respective age and sex quota were already full;

N=1879

Screen outs:

Respondents did not fit one of the pre-specified subgroups regarding FDRs with or without cancer;

N=528

Drop-outs:

Respondents started to complete the questionnaire
but did not finish it;

N=224

Respondents included in this analysis:

N=924

Included respondents overall:

N=1012

Respondents overall:

N=3643
